# CD5+ Primary Cutaneous Diffuse Large B-Cell Lymphoma, Leg Type, Presenting as an Asymptomatic Nodule

**DOI:** 10.3390/hematolrep15030053

**Published:** 2023-09-01

**Authors:** Amy Xiao, Colleen J. Beatty, Sonal Choudhary, Oleg E. Akilov

**Affiliations:** 1 School of Medicine, University of Pittsburgh, Pittsburgh, PA 15260, USA; xiaoa2@upmc.edu; 2 Department of Dermatology, University of Pittsburgh, Pittsburgh, PA 15261-2109, USA; beattycj@upmc.edu (C.J.B.); choudharys@upmc.edu (S.C.)

**Keywords:** cutaneous lymphoma, leg type, nodule, leg, B cell

## Abstract

Primary cutaneous diffuse large B-cell lymphoma, leg type (PCDLBCL-LT), is a rare and aggressive variant of primary cutaneous lymphoma that typically expresses B cells as well as MUM1/IRF4, BCL2, and FOXP1, whereas BCL6 may be present or undetectable. We present a case of CD5+ PCDLBCL-LT presenting as a 6 mm pink-bluish nodule on the mid-left thigh, which was concerning for basal cell carcinoma. The histological examination reveals the presence of an intradermal proliferation of large, atypical CD5+, CD20+ BCL2+, BCL6+, MUM-1+, and Cyclin-D1+ lymphocytes in a nodular, diffuse interstitial and perivascular distribution. Because the patient presented with a small, single nodule, the systemic treatment of multiagent chemotherapy was avoided and localized electron beam radiation therapy with rituximab was initiated instead, achieving complete response. Early identification of PCDLBCL-LT is key for maximal therapeutic benefit and prognosis; it is important to consider PCDLBCL-LT on the differential when evaluating small, single nodules on the lower extremities of elderly patients.

## 1. Introduction

Primary cutaneous diffuse large B-cell lymphoma, leg type (PCDLBCL-LT), is a rare and aggressive variant of primary cutaneous lymphoma characterized by multiple nodules in the lower extremities, most commonly found in elderly females [1,2,3,4,5,6,7]. We present the seventh case of CD5+ PCDLBCL-LT to our knowledge, in an otherwise asymptomatic 89-year-old woman who initially presented with a small, 6 mm pink/purple nodule on her leg.

## 2. Case

**Figure 1:**
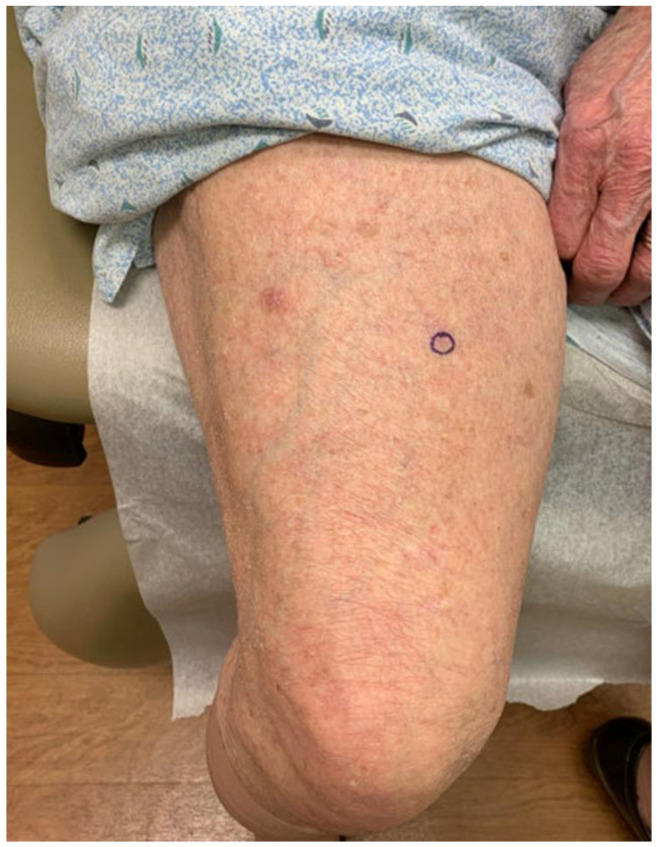
Primary cutaneous diffuse large B-cell lymphoma, leg type on left thigh.

**Figure 2:**
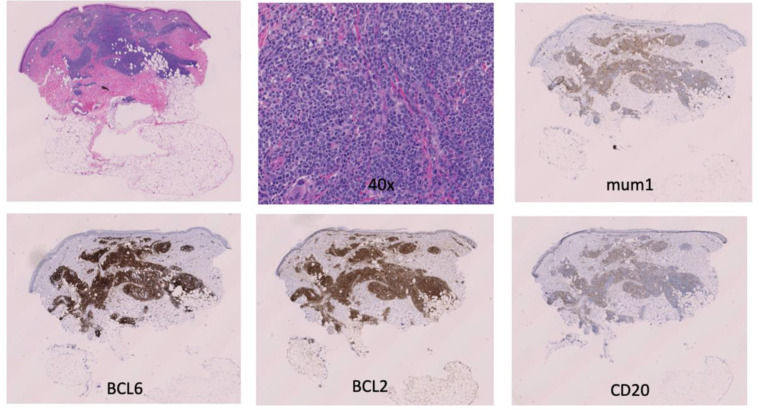
Primary cutaneous diffuse large B-cell lymphoma, leg-type: H & E, 10×, 40×, mum1, BCL6, BCL2, CD20.

An 89-year-old woman with a remote history of bilateral breast adenocarcinoma s/p radiation and chemotherapy presented with a 6 mm pink-bluish nodule on her mid left thigh (Figure 1). The nodule was concerning for basal cell carcinoma and was removed by shave biopsy. The histological examination reveals the presence of an intradermal proliferation of large, atypical CD5+, CD20+ BCL2+, BCL6+, MUM-1+, and Cyclin-D1+ lymphocytes in a nodular, diffuse interstitial and perivascular distribution (Figure 2). The cells themselves are hyperchromatic with coarse chromatin, angulated nuclei, and vacuolated cytoplasms of centroblast and immunoblast morphology. The Ki67 proliferative index was >50% in the cells of interest. Clonality was positive for IgH gene rearrangement. MYC, BCL2, CCND1, CEP 8, and IGH/MYC FISH studies were negative for gene rearrangement. BCL6 FISH studies were negative for gene rearrangement and showed 10% of cells with one extra signal for BCL6. CD21, CD23, and EBER were negative. These findings were compatible with large B-cell lymphoma. While the 18FDG-PET/CT scan did not show any systemic involvement, a subthreshold uptake in a tiny cutaneous lesion in the lower medial right thigh was noted. Flow cytometry analysis of the peripheral blood was performed, and no clonal expansion of B cells was found. Thus, the diagnosis of PCBCL-LT was rendered. Since the patient had a single nodule, the decision was made to avoid multiagent chemotherapy. Instead, localized electron beam radiation therapy was initiated and followed by four weekly doses of rituximab, and the patient achieved a complete response sustained for ten months and ongoing.

## 3. Discussion

PCDLBCL-LT frequently manifests by multiple tumors at the beginning. It was very unusual to find that a small singular nodule resembling basal cell carcinoma or dermatofibroma came back as PCDLLBCL-LT. Moreover, the expression of CD5 is uncommon in PCDLBCL-LT. We found seven documented cases of CD5+ PCDLBCL-LT in the literature [8,9,10,11,12,13,14] (Table 1).

In addition to the B-cell-associated antigens, the tumor cells of PCDLBCL-LT are often positive for Bcl-6 and Bcl-2 [14]. Of the existing cases in the table above, Bcl-2 is positive in three [8,9,13,14]. One case had both Bcl-6 and Bcl-2 positivity [9]. Another had positivity in BCL2, BCL6, and c-MYC. The translocation in those three genes is known as “triple hit” status, associated with poor prognosis [14]. While the absence of triple hit status is a favorable prognosticator, the prognostic significance of CD5 positivity is poorly characterized. Of the patients with CD5+ PCDLBCL-LT without systemic involvement at the time of diagnosis, who underwent treatment, complete response was achieved for all patients, indicating better outcomes than with CD5- PCDLBCL-LT (Table 1).

The differential diagnosis for our case, besides basal cell carcinoma and dermatofibroma, includes primary cutaneous follicle center and mantle cell lymphomas. Mantle cell lymphoma is also CD5+ and can present as a cutaneous nodule. While the presence of IGH gene rearrangement indicates a clonal population, it would be beneficial to examine for the CCND1-IGH rearrangement, as it serves as a distinctive hallmark of the disease [15]. Partial positivity of CD10, as in our case, may be seen in follicle center lymphomas [8]. However, the histologic examination did not identify a follicular pattern, and our case was CD5+ and IgM+, which are both negative in follicle center lymphoma [9]. While there were also centroblast-like cells, which are seen in follicle center lymphoma, the immunophenotype of our case was more like that of PCDLBCL-LT. LEF-1, found in monoclonal B-cell lymphocytosis; FOXP1, which is expressed in B cells; as well as BCL2, which is typically found in PCDLBCL-LT, were positive and so clinicopathologic features were felt to be more convincing for PCDLBCL-LT [1,2,3,4,5].

Primary cutaneous diffuse large B-cell lymphoma, leg type, is a rare variant of primary cutaneous B-cell lymphoma with a poor prognosis. Thus, early identification of PCDLBCL-LT is beneficial to the patient in terms of therapeutic benefit and prognosis. PCDLBCL-LT should be on the differential diagnosis when evaluating single papules or nodules on the lower extremities, including basal cell carcinoma, Merkel cell carcinoma, dermatofibroma, and dermatofibrosarcoma protuberans, among others.

## Figures and Tables

**Table 1:** Reported cases of CD5+ PCDLBCL-LT.

Source	Age, Gender, Clinical Description	Histopathology	Histological Phenotype	Treatment and Outcome
Current case	89 y/o F 6 mm pink-bluish nodule on mid left thigh	nodular infiltrate of medium-to-large, atypical CD5, BCL6, BCL2, LEF-1, and CD43 + lymphocytes in the dermis	CD5+, BCL-6+, BCL-2+, CD43+, LEF-1+ CD21−, CD23-	Localized electron beam radiation and rituximab. CR for 11 months
Hembury et al. [8]	64 y/o M Breast nodule	N/A	CD5+, BCL-2+, C20+ BCL-6−, CD10−, p53−	Chemotherapy CR for 197 months
Papoudou-Bai [9]	71 y/o F nodule of left thigh with CT showing diffuse lesions c/f DLBCL of the central nervous system	diffuse dermal infiltration by large lymphoid cells	CD5+, CD20+, CD79a+, MUM1/IRF4+, BCL6+, BCL2+, cytoplasmic IgM/λ + CD3−, CD56−, CD23−, CD21−, CD10−, CD30−, cyclin D1−, CD68−, lysozyme−, myeloperoxidase−, CD34−	Methotrexate Death from progressive disease and epileptic seizures
Tanaka et al. [10]	45 y/o F Several subcutaneous nodules, 1 to 3 cm, on the right arm and trunk	N/A	CD5+, CD19+, CD20+, CD22+, CD44+, HLA-DR+, CD7+, and surface IgM K chains+ Absent Biomarkers N/A	Chemotherapy CR for 14 months
Takahashi et al. [11]	71 y/o F Multiple firm subcutaneous nodules ranging from 0.3 to 2.0 cm in diameter, in bilateral lateral chest, abdomen, back, and buttocks	infiltration of atypical large cells with distinct nucleoli	CD5+, CD19+, CD20+, CD25+, IgM+ and λ-chain+ CD10−, CD23−, ĸ-chain−	CHOP CR for 7 months
Goto et al. [12]	52 y/o F tender subcutaneous nodules on the neck, forearms, trunk, and legs	atypical diffuse large cells infiltrating subcutaneous tissues	CD5+, CD20+, CD79a+ CD10−, cyclin D1−, TdT−	R-CHOP/APBSCT CR for 24 months
Dargent et al. [13]	79 y/o F skin lymphoma involving the right wrist area	numerous centroblasts infiltrating both the dermis and the subcutaneous tissue	CD5+, CD20+, CD79a+, BCL-2+ Anaplastic large cell lymphoma kinase (ALK)−, CD10−, CD23−, CD30−, CD43−, Bcl-6−, cyclin D1−, p53−, p16INK4a−	Complete surgical excision CR for 19 months
Sun, et al. [14]	69 y/o F multiple red nodules on the right lower limb	Heterogenous, large, infiltrating cells with lightly stained cytoplasm and irregularly large, vacuolar nuclei, centroblasts, and immunoblasts	CD5+, CD20+, Pax-5+, BCL-2+, BCL-6+, MUM-1+, c-myc+, Ki-67+ Absent biomarkers N/A	Declined treatment. Outcome N/A

Abbreviations: CHOP, Cyclophosphamide, Adriamycin, Vincristine, and Prednisolone; NA, not available; R-CHOP, Rituximab-CHOP; CR, complete remission; APBSCT, autologous, peripheral blood stem cell transplantation.

## Data Availability

No new data were created.
